# Application of a Low-Level Laser Therapy and the Purified Protein from Natural Latex (*Hevea brasiliensis*) in the Controlled Crush Injury of the Sciatic Nerve of Rats: A Morphological, Quantitative, and Ultrastructural Study

**DOI:** 10.1155/2013/597863

**Published:** 2013-07-02

**Authors:** Fernando José Dias, João Paulo Mardegan Issa, Mamie Mizusaki Iyomasa, Joaquim Coutinho-Netto, Ricardo Alexandre Junqueria Calzzani, Daniela Mizusaki Iyomasa, Luiz Gustavo Sousa, Sonia Regina Yokomizo de Almeida, Diego Pulzatto Cury, Ii-sei Watanabe

**Affiliations:** ^1^Department of Anatomy, Institute of Biomedical Sciences, University of Sao Paulo, 2415 Avenida Professor Lineu Prestes, 05508-900 Sao Paulo, SP, Brazil; ^2^Department of Morphology, Physiology and Basic Pathology, Faculty of Dentistry of Ribeirao Preto, University of Sao Paulo, S/N Avenida do Café, 14040-904 Ribeirao Preto, SP, Brazil; ^3^Department of Pharmacology, Faculty of Medicine of Ribeirao Preto, University of Sao Paulo, 3900 Avenida Bandeirantes, 14049-900 Ribeirao Preto, SP, Brazil; ^4^Dental School, University of La Frontera, 1145 Avenida Francisco Salazar, 01145 Temuco, Chile

## Abstract

This study analyzed the effects of a low-level laser therapy (LLLT, 15 J/cm^2^, 780 nm wavelength) and the natural latex protein (P1, 0.1%) in sciatic nerve after crush injury (15 Kgf, axonotmesis) in rats. Sixty rats (male, 250 g) were allocated into the 6 groups (*n* = 10): CG—control group; EG—nerve exposed; IG—injured nerve without treatment; LG—crushed nerve treated with LLLT; PG—injured nerve treated with P1; and LPG—injured nerve treated with LLLT and P1. After 4 or 8 weeks, the nerve samples were processed for morphological, histological quantification and ultrastructural analysis. After 4 weeks, the myelin density and morphological characteristics improved in groups LG, PG, and LPG compared to IG. After 8 weeks, PG, and LPG were similar to CG and the capillary density was higher in the LG, PG, and LPG. In the ultrastructural analysis the PG and LPG had characteristics that were similar to the CG. The application of LLLT and/or P1 improved the recovery from the nerve crush injury, and in the long term, the P1 protein was the better treatment used, since only the application of LLLT has not reached the same results, and these treatments applied together did not potentiate the recovery.

## 1. Introduction

Peripheral nerves are extensions from neurons that are located in columns and spinal cord ganglia, and the peripheral portions are dependent on the continuity and integrity of the central portions [1].

Approximately 4.5% of injuries to soft tissue and 5% of open wounds in the extremities that are due to accidents result in an impairment of peripheral nerves that requires surgical repair [2]. The treatment of peripheral nerve injuries remains a challenge, despite advances in the understanding of nerve regeneration in microsurgical techniques, and the functional recovery from postperipheral nerve repair remains unsatisfactory [3, 4]. The consequences include injury and retrograde axonal degeneration of neurons followed by a very slow regeneration [4]. Nerve regeneration can be studied using several experimental models, but the crush model is advantageous because it does not involve section and suture variables [5].

Many studies that have evaluated the low-level laser therapy (LLLT) as a treatment for peripheral nerve injury have observed several changes that are obtained with this therapy [6], such as improvement in function, neuromuscular activity, and evocation [7–11]; thus, it reduces neuropathic pain [12], accelerates migration, cell growth, and nerve fiber sprouting in cell cultures [8], changes the activity of nerve cells, induces the upregulation of several neurotrophic growth factors and extracellular matrix proteins that support neurite outgrowth [13], and stimulates Schwann cell proliferation and the expression of NGF [14]. Specifically, in the crush model using the LLLT (660 and 780 nm wavelength, 10 and 60 J/cm^2^), which is irradiated on the sciatic nerve of rats, treatment restored the myelin sheath and cross-sectional area of the nerve [15]. These data became the basis for clinical research evaluating the effectiveness of LLLT treatment in patients suffering from lesions on peripheral nerves [10].

A new protein derived from the rubber tree (*Hevea brasiliensis*), named P1 protein, was identified at the Laboratory of Neurochemistry of Faculty of Medicine of Ribeirao Preto, University of Sao Paulo [16]. This protein stimulates angiogenic activity, cell adhesion, extracellular matrix formation, substitution, and tissue regeneration as well as improves wound healing in skin tissue, the pericardium, esophagus, abdominal wall, tympanum, blood vessels, and connective tissue of eye without producing signs of hypersensitivity [17, 18]. In addition to these results, the P1 protein also acted on dermal ulcers, increasing vascular permeability and improving the healing process [18], and the retina, where it increased neovascularization [19]. In mineralized tissues, the P1 protein facilitates the reconstitution of bone defects by vascularizing the collagen matrix [20]. To simulate clinical conditions in rats, a biomembrane containing the P1 protein was used as a neurotube (axonal growth guide) in a study of sciatic nerve resection. This material improved the quality of nerve regeneration, nerve impulse conduction, and the functional gait of the animals [21]. 

Thus, the present study aims to evaluate the effects of the LLLT along with the new biomaterial, the P1 protein, in the treatment of controlled nerve crush injury using morphological, quantitative, and ultrastructural analyses.

## 2. Material and Methods

### 2.1. Animals

Sixty Wistar rats (♂, 250–300 g) were allocated into six groups (*n* = 10). Animals were housed in polyethylene boxes with 5 animals each in rooms with controlled temperature (22–24°C) and dark/light cycles of 12 h. They were fed with chow and water *ad libitum* and were not subjected to unnecessary stress. The study protocol was approved by the local Ethics Committee (Protocol no. 078/2010) according to international laws of animal use.

### 2.2. Nerve Injury

 Animals were anesthetized with ketamine and xylazine at dosages of 75 and 10 mg/kg, respectively, and after the left sciatic nerve was exposed, they were positioned on a stainless steel base coupled to a EMIC universal testing machine (EMIC-DL2000, Paraná, Brazil). The left sciatic nerve crush was performed according to the method of a previous study [22]. The site of injury was standardized based on two bone processes from the iliac crest easy to locate (upper and inferior ventral spine). The skin incision (2 cm approximately) was made perpendicularly and 2 cm toward at the middle region between these points, then the muscle fascia was ruptured over the region of anterior belly of the biceps femoris and gluteus maximus, and these muscles were divulsed, not requiring incision, and finally the nerve was exposed to injury. To collect the nerve samples we followed the same anatomical references. The weight applied on the sciatic nerve was a constant force of 15 kgf for 10 minutes, crushing a circular area of approximately 0.28 cm^2^ (0.6 cm diameter) totaling 5.2 MPa. After the injury, the nerve was repositioned, the skin was sutured, and antiseptic, anti-inflammatory, and antibiotic medications were applied to prevent further complications.

### 2.3. Low-Level Laser Therapy**—**LLLT

 The Twin Laser apparatus (MM Optics, Sao Carlos, Brazil) apparatus was used for laser irradiation. The application of LLLT was performed in a transcutaneous way in order to simulate clinical use. Three points of application were chosen following the path of the sciatic nerve for better distribution of energy. The application sites were determined by the scar on the skin related to surgical access to the sciatic nerve. The three points were 1 point proximal to the lesion area, 1 point in the damaged area, taking care to move the scar pulling up the skin, so interference will not occur in the scar tissue absorption and scattering of LLLT, and finally 1 point distal region of the scar. During the laser application, a contention was used to immobilize the animals to avoid the use of local or general anesthesia. The parameters for laser irradiation are listed in Table 1.

### 2.4. Natural Protein from Latex (*Hevea brasiliensis*)—P1 Protein

The vehicle used to deliver the P1 protein from *Hevea brasiliensis* was the hyaluronic acid [23]. The hyaluronic acid (Nikkol, Japan), isolated from gram-negative bacteria, was used at a final concentration of 1% with the 0.1% P1 protein. The mixture was inserted using a micropipette (100 *μ*L) *in situ* after injury and just before the suture. Hyaluronic acid and P1 protein were provided by the Laboratory of Neurochemistry, Faculty of Medicine of Ribeirao Preto, University of Sao Paulo.

### 2.5. Experimental Groups

 The current study evaluated six groups (*n* = 10) at four (*n* = 5) and eight weeks (*n* = 5) after the injury, and the protocols for each group are described below. 

Control group (CG) animals were anesthetized and kept lying in a lateral position for 10 minutes. For the exposed group (EG), the sciatic nerve was exposed and positioned on the lesion support for 10 minutes. In the injured group (IG), the sciatic nerve was exposed and crushed. The LLLT group (LG) had the sciatic nerve exposed and crushed, and then the animals were submitted to the LLLT irradiation protocol. For the protein group (PG), the sciatic nerve was exposed and crushed, and then the P1 protein was applied on the lesion site. In the laser and protein group (LPG), the sciatic nerve was exposed and crushed, the P1 protein was applied on the injury site, and then the animals were submitted to the laser protocol. 

### 2.6. Samples Processing and Analyses

 Four or 8 weeks following the nerve injury, the animals were anesthetized (ketamine and xylazine at dosages of 75 and 10 mg/kg, resp.) and subjected to intracardiac perfusion with a solution containing 2.5% glutaraldehyde and 2% paraformaldehyde in 0.1 M sodium phosphate buffer [24]. To process the sciatic nerve samples for morphological analysis by light microscopy, the samples were embedded in “Leica Historesin,” cut with glass knives (3 *μ*m), mounted on slides, and stained with neutral red and black Sudan to visualize the myelin sheaths of nerve fibers. The histological analyses were performed using a DM4000 B Leica microscope with the EC3 Leica camera. To process the nerve samples for analysis using transmission electron microscopy, the samples were fixed in 2.5% glutaraldehyde for 2 hours, post-fixed in 1% OsO_4_ at 4°C for 2 h, dehydrated in an ethanol series and propylene oxide, and embedded in Spurr resin. Finally, ultrathin sections (90 nm) were mounted on 200 mesh grids, counterstained with uranyl acetate and lead citrate, and examined under a JEOL 1010 electron microscope operating at 80 kV at the Institute of Biomedical Sciences, University of Sao Paulo.

### 2.7. Quantitative Analysis

**Figure 1:**
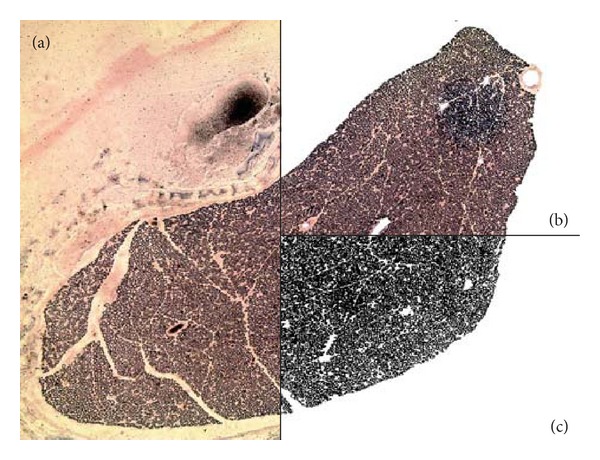
Myelin density quantification: (a) original sciatic nerve photomicrograph; (b) photomicrograph of the sciatic nerve with the peripheral and blood capillaries areas cleared; (c) photomicrograph in binary (black and white) used during quantification.

 The quantitative histological analysis of the myelin consisted of quantifying the number of pixels. In the original images (Figure 1(a)), the external and the capillaries areas have been erased (Figure 1(b)) using the software Photoshop CS, and this procedure was necessary since this dark staining in particular areas of the erythrocytes could be interpreted as myelin. The next step is to convert the cleaned images into binary form (black and white) (Figure 1(c)), which was performed using the ImageJ 1.45 s software, with the following commands “process/binary/make it binary,” or if you prefer to use Photoshop CS software commands “image/adjust/threshold,” in this case it is necessary to choose a threshold point (1–255), which once chosen, this value should be respected in all images analyzed; in this study the images were transformed into binary by Photoshop CS method with the threshold point of 128. The binary images in ImageJ software with the following command “plugins/analyze/area calculator” (area calculator plugin, free download at http://rsb.info.nih.gov/ij/plugins/area.html/, not included in original plugin pack of ImageJ) provide the area of the dark region in pixels^2^ representing myelin; if the program was calibrated to the scale of the microscope used in the capture of the images, it is possible to obtain the same area in *μ*m^2^. For the realization of area ratio of myelin (myelin area/nerve area) it was also necessary to measure the cross-sectional area of the sciatic nerve, which was obtained from the original images (Figure 1(a)) using the command “Polygon selections” encircling the region of the nerve and then the command “analyze/measure.” The ratio was obtained by dividing the first value obtained in relation to the cross-sectional area of the nerve; these data were compared between groups. For the analysis of capillary density, blood vessels were counted and then the ratio of vessels/nerve area (capillaries/mm^2^) was compared among the groups. Statistical analysis of the quantitative analyses was performed using the SPSS 17.0 software, employing an analysis of variance (ANOVA, *P* < 0.05).

## 3. Results

### 3.1. Morphological Analysis

**Figure 2:**
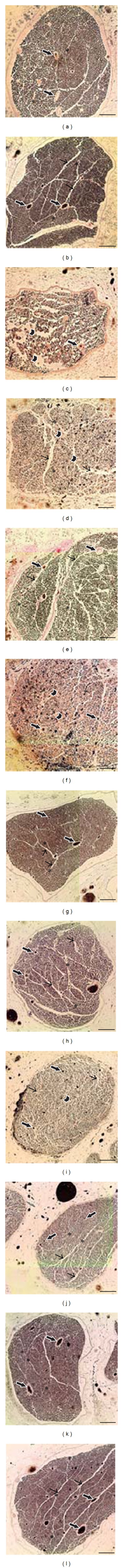
Light microscopy morphology—(a) CG—4 weeks—normal bundles of nerve fibers (*) and blood capillaries (arrows) (mag: ×10, bar: 100 *μ*m). (b) EG—4 weeks—bundles of nerve fibers (*), blood capillaries (arrows), and the perineurium area (thin arrows) (mag: ×10, bar: 100 *μ*m). (c) IG—4 weeks—spaces (arrowheads) among nerve fibers (*) containing an amorphous substance and blood capillaries (arrow) (mag: ×10, bar: 100 *μ*m). (d) LG 4-weeks—bundles of nerve fibers (*) surrounded by spaces (arrowheads) and the perineurium (thin arrow) (magnification: ×10 bar: 100 *μ*m). (e) PG—weeks—the nerve fibers (*), the perineurium (thin arrows), and blood capillaries (arrows) are noted (mag: ×10, bar: 100 *μ*m). (f) LPG—4 weeks—reveals the bundles of nerve fibers (*), the space between the fibers (arrowheads) and capillaries (arrows) (mag: ×10, bar: 100 *μ*m). (g) CG—8 weeks—normal nerve fibers (*) divided into bundles by perineurium (thin arrow) and blood capillaries (arrows) (mag: ×10, bar: 100 *μ*m). (h) EG—8 weeks—bundles of nerve fibers (*), the perineurium region (thin arrows), and blood capillaries (arrows) (mag: ×10, bar: 100 *μ*m). (i) IG—8 weeks—nerve fibers (*), capillaries (arrows), the perineurium (thin arrows), and the spaces between the fibers (arrowhead) (mag: ×10, bar: 100 *μ*m). (j) LG—8 weeks—bundles of nerve fibers (*), the perineurium region (thin arrows), and blood capillaries (arrows) (mag: ×10, bar: 100 *μ*m). (k) PG—8 weeks—bundles of nerve fibers (*) and blood capillaries (arrows) similar to the CG (mag: ×10, bar: 100 *μ*m). (l) LPG—8 weeks—nerve fibers (*), the perineurium (thin arrows), and blood capillaries (arrows) similar to the CG (mag: ×10, bar: 100 *μ*m).

 Histological analysis after 4 weeks from the start of the experiment revealed normal morphology in the CG (Figure 2(a)) and EG (Figure 2(b)) without changes in the nerve fibers with different diameters, which were arranged for conjunctive wraps and the presence of capillaries of different sizes distributed through the cross-sectional area of the sciatic nerve. However, the IG (Figure 2(c)) had significant changes in morphologic characteristics, such as the loss of myelinated nerve fiber density, myelin fibers of reduced and uniform size, the appearance of spaces between nerve fibers containing an amorphous substance, disorganization of the perineural region, and changes in blood capillaries, which reflected structural changes of the sciatic nerve in that group. The other groups that were analyzed 4 weeks after nerve injury had marked morphological characteristics compared to the IG. The LG (Figure 2(d)) and LPG (Figure 2(f)) had similar morphological characteristics, in which the internal contents of the sciatic nerve were more organized and had reduced space between the nerve fibers compared to the IG; however, these groups still had a loss of nerve fiber density and altered perineural areas in this period of analysis. The PG (Figure 2(e)) had characteristics that were closer to the CG, with a better organization of the internal components of the sciatic nerve and a greater density of nerve fibers; however, the results still indicated a loss of quality in these characteristics compared to the control group samples.

Eight weeks after the nerve crush injury, the morphological characteristics of the CG (Figure 2(g)) and EG (Figure 2(h)) were normal, as observed in the previous period of analysis, and only a slight spacing of nerve fibers was observed in the EG. Again, the IG (Figure 2(i)) revealed density loss for nerve fibers, but in this period of analysis, the spaces between the fibers were no longer present and the internal organization was better compared with the IG samples observed 4 weeks after the injury. The LG (Figure 2(j)) had improved morphology compared with the IG after 8 weeks and with the LG after 4 weeks of nerve injury because the density and organization of nerve fibers were higher in this group. In this period of analysis, the PG (Figure 2(k)) and LPG (Figure 2(l)) revealed different characteristics, corresponding to the groups analyzed in the fourth postoperative week, and the observed features at 8 weeks resemble the characteristics of the CG and EG.

### 3.2. Ultrastructural Analysis—Transmission Electron Microscopy

**Figure 3:**
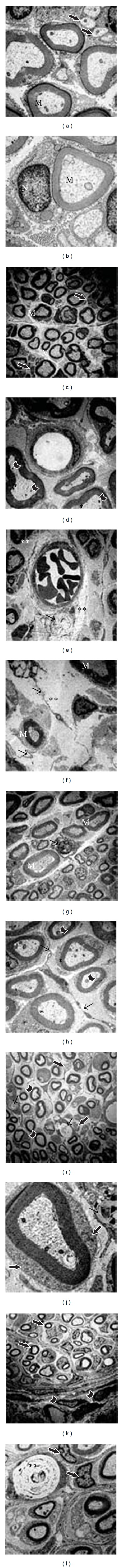
Transmission electron microscopy after 4 weeks of nerve injury: (a) CG—normal myelinated (M) and unmyelinated (arrows) fibers (mag: ×6.000). (b) CG—detailed image of the Schwann cell nucleus (N) involving a myelin nerve fiber (M) (mag: ×20.000). (c) EG—grouping of myelinated fibers (M) and a few unmyelinated fibers (arrows) (mag: ×2.000). (d) EG—detailed image of the axoplasm of the myelin fiber (*) with mitochondria (arrowheads) (mag: ×12.000). (e) IG—area of degeneration of nerve fibers (thin arrows) below the capillary blood and spaces among the nerve fibers (**) (mag: ×3.000). (f) IG—detailed image of the degenerating nerve fibers area (**), intact myelinated fibers (M), and linear structures that indicate Schwann cell degeneration (thin arrows) (mag: ×6.000). (g) LG—myelinated fibers (M) and degenerated Schwann cells (thin arrow) (mag: ×2.500). (h) LG—detailed image of the axoplasm of intact myelinated fibers (*) containing mitochondria (arrowheads) and remnants of Schwann cells (thin arrow) (mag: ×6.000). (i) PG—myelinated (arrowheads) and unmyelinated fibers (arrows) amid areas of degeneration (thin arrows) (mag: ×1.800). (j) PG—detailed image of myelinated fibers enveloped by Schwann cell cytoplasm containing mitochondria (arrows) and degeneration adjacent area (thin arrow) (mag: ×7.000). (k) LPG—grouping of myelinated fibers (arrows) and adjacent fibroblasts (arrowheads) in the perineural region (mag: ×1.800). (l) LPG—detailed image of a degenerated area among nerve fibers (**) adjacent to Schwann cells (arrows) (mag: ×5.600).

In the fourth postoperative week, the CG had no alterations in the sciatic nerve, which contained both myelinated and unmyelinated nerve fibers of different sizes arranged in groups, and most of the fibers had thick myelin sheaths (Figure 3(a)). In the axoplasm, the mitochondria and neurofilaments could be observed, and collagen fibers were present in the endoneurial region. Additionally, the Schwann cells had a normal appearance and included nerve fibers (Figure 3(b)). The EG had normal characteristics, as myelin fibers of many diameters and shapes in separate bundles and few unmyelinated fibers were visible (Figure 3(c)). Myelinated nerve fibers were surrounded by Schwann cell cytoplasm. In the axoplasm of nerve fibers, neurofilaments and mitochondria were observed (Figure 3(d)). In the IG, myelin fibers were spaced (Figure 3(e)), as noted in the amorphous endoneurium areas. There were areas of degeneration of nerve fibers and electron-dense structures in the shape of lines that corresponded to the residues of Schwann cells (Figure 3(f)). In the samples obtained from the LG, there were unmyelinated and myelinated fibers of varying diameters, with smaller spacing between the fibers compared to the IG; however, there were still visible characteristics of nerve degeneration (Figure 3(g)). The axoplasm encloses neurofilaments and mitochondria, and the Schwann cells surround nerve fibers. However, there were signs of degeneration, such as linear structures corresponding to the remains of Schwann cells (Figure 3(h)). The PG revealed myelinated and unmyelinated fibers with homogeneous diameters organized into bundles, and there were still regions of nerve degeneration but with smaller areas compared to the IG and LG (Figure 3(i)). The details of the myelin fiber revealed that it was surrounded by Schwann cell cytoplasm, containing mitochondria adjacent to the degenerated fibers (Figure 3(j)). In the LPG, there were many myelinated nerve fibers arranged in bundles and there was little variation in their diameters, and unmyelinated fibers were rarely observed (Figure 3(k)). In this group, areas of the degeneration of nerve fibers were observed in the midst of normal nerve fibers (Figure 3(l)).

**Figure 4:**
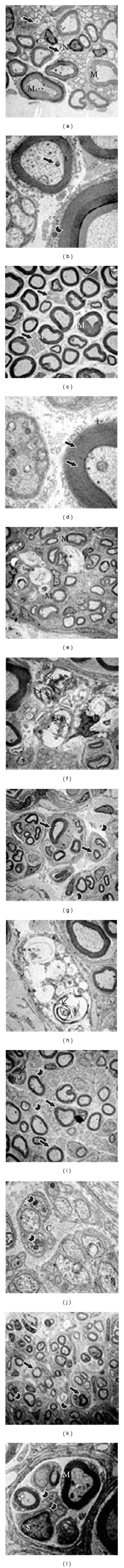
Transmission electron microscopy after 8 weeks of nerve injury: (a) CG—general view of the sciatic nerve fibers with myelin (M), groups of unmyelinated fibers (arrows), and Schwann cells with evident nucleus (N) (mag: ×3.000). (b) CG—detailed image of mitochondria in the axoplasm of a myelinated fiber (arrow) and in the cytoplasm of Schwann cells (arrowhead) (mag: ×20.000). (c) EG—myelinated fibers (M) and a grouping of unmyelinated fibers (arrows) (mag: ×3.000). (d) EG—detailed image of the neurofilaments of the axoplasm (*) and the lamellae of myelin (arrows) (mag: ×30.000). (e) IG—degenerated areas (**) among the intact myelin fibers (M) (mag: ×3.000). (f) IG—Details of an area of degeneration of nerve fibers in the presence of myelin debris (thin arrows) (mag: ×5.000). (g) LG—myelinated (arrows) and unmyelinated fibers (arrowheads) arranged in bundles (mag: ×2.500). (h) LG—degenerated area of nerve fibers (**) containing myelin debris (thin arrows)—(mag: ×6.000). (i) PG—grouping of myelinated (arrows) and unmyelinated (arrowheads) nerve fibers, and an area of degeneration (thin arrow) (mag: ×2.500). (j) PG—detailed image of groupings of unmyelinated fibers with mitochondria (arrowheads) in axoplasm (*) and collagen fibers of the endoneurium (C) (mag: ×20.000). (k) LPG—general view of bundles of myelinated fibers (arrows) and an area of degeneration (arrowheads) (mag: ×1.800). (l) LPG—detailed image of a bundle of nerve fibers reveals myelinated and (M) unmyelinated fibers (arrowheads) and Schwann cells (S) (mag: ×5.600).

Eight weeks after the injury, the CG had characteristics similar to those observed in the control group that was analyzed after four weeks, as the groups of myelinated and unmyelinated fibers were present in various sizes and shapes and Schwann cells were present surrounding the fibers (Figure 4(a)). The myelinated fibers adjacent to one group of unmyelinated fibers are shown in detail in the endoneurial area that contains collagen fibers and the cytoplasm of Schwann cells with mitochondria (Figure 4(b)). In the EG, myelin fibers and large amounts of unmyelinated fibers of varying diameters were present (Figure 4(c)). In the axoplasm of unmyelinated and myelinated fibers, neurofilaments and mitochondria were revealed, and the lamellar organization of the myelin sheath was also observed (Figure 4(d)). Characteristics of degeneration were not observed in these first two groups during this analysis period. The IG presented the presence of nerve fibers that were closer to each other compared to the IG 4 weeks after the injury; however, the presence of large areas of nerve fibers degeneration located between intact fibers was visible (Figure 4(e)). Myelin debris could be observed in the areas of nerve fiber degeneration (Figure 4(f)). In the LG, one could observe that the myelinated nerve fibers and unmyelinated diameters and shapes were arranged in bundles, with large spaces between nerve fibers and structures of the linear remnants of Schwann cells (Figure 4(g)). Additionally, the myelin debris in an area of degeneration was surrounded by intact myelinated and unmyelinated fibers (Figure 4(h)). The PG had numerous unmyelinated and myelinated fibers of varying diameters, with few areas of degeneration between the intact fibers (Figure 4(i)). A more detailed view of the myelinated fiber the sheath revealed normal characteristics around the axoplasm, enveloped by a Schwann cell, and collagen fibers were observed in the endoneurial region (Figure 4(j)). The LPG revealed the presence of myelinated and unmyelinated fibers that were arranged in bundles and regions of nerve degeneration with the Schwann cell cytoplasm having a dense appearance; additionally, some areas of fiber degeneration were still observable (Figure 4(k)). The grouping of myelinated and unmyelinated nerve fibers of various diameters could be observed in detail, and the axoplasm contained neurofilaments and mitochondria along with the presence of a Schwann cell with an evident nucleus (Figure 4(l)).

### 3.3. Quantitative Analyses

**Figure 5:**
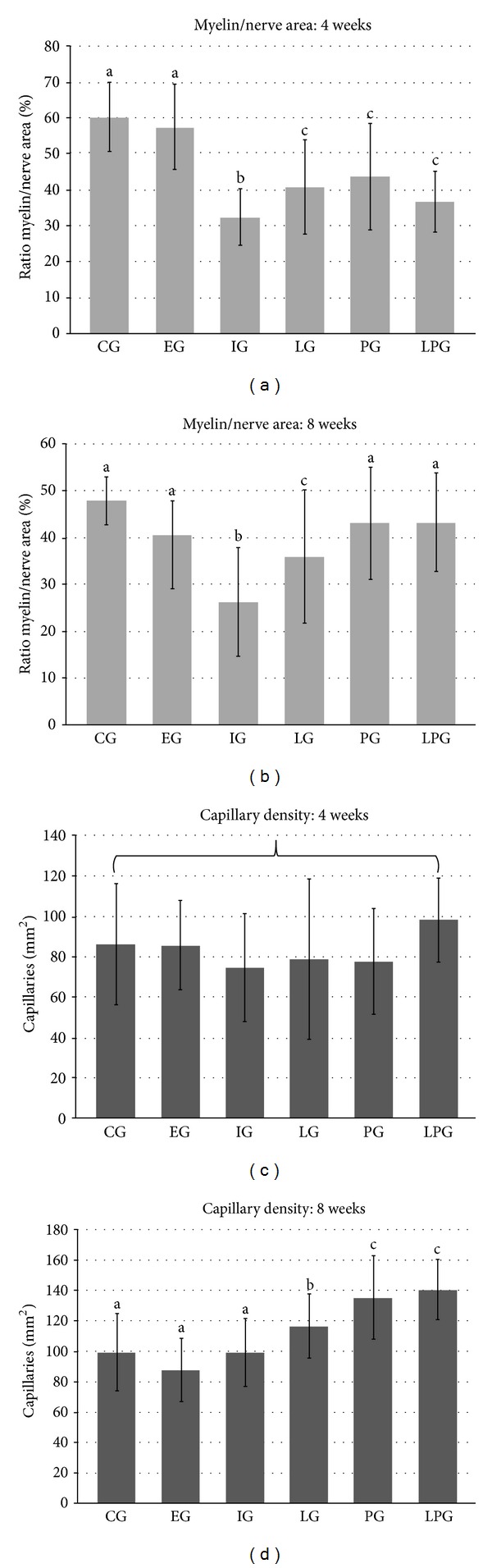
(a) Chart 1—ratio myelin/nerve area, 4 weeks after injury. (b) Chart 2—ratio myelin/nerve area, 8 weeks after injury. (c) Chart 3—capillary density, 4 weeks after injury. (d) Chart 4—capillary density, 8 weeks after injury.

The histological quantification in the fourth postoperative week (Table 2) revealed that the ratio of the myelin/cross-sectional area of the sciatic nerve (Figure 5(a)) in the CG (60.28 ± 9.66) and EG (57.55 ± 11.86) was statistically similar and higher than in the other groups. The myelin/cross-sectional area ratios of the LG (40.81 ± 13.07), PG (43.72 ± 14.83), and LPG (36.64 ± 8.32) were similar and had values significantly higher than the IG (32.37 ± 7.78), which was the group with the lowest value in this analysis period. The analysis of the blood capillary density data (number of capillaries/mm^2^) (Figure 5(b)) from the six study groups 4 weeks after the injury revealed no significant differences (CG—86.03 ± 30.08; EG—85.69 ± 22.07, IG—75.45 ± 26.62, LG—78.83 ± 39.67; PG—77.80 ± 26.10; and LPG—98.16 ± 20.87).

Data from the histological quantification 8 weeks after nerve injury are shown in Table 3, and analysis of the myelin/nerve area ratio (Figure 5(c)) revealed that the CG (47.85 ± 5.06), EG (40.60 ± 7.19), PG (43.04 ± 12.00), and LPG (43.19 ± 10.55) were statistically similar and had higher values of this parameter compared to the other groups. Thus, the LG (35.89 ± 14.25) had a lower value for this parameter compared to the previous group; however, it was higher than the IG (26.14 ± 11.61). The capillary density values (number of capillaries/mm^2^) (Figure 5(d)) 8 weeks after causing the nerve lesion revealed that the CG (99.22 ± 25.29), EG (87.60 ± 20.88), and IG (99.12 ± 22.56) were similar, as they had the lowest values of this parameter. While the LG (116.59 ± 21.24) had significantly higher values compared to these three groups, its value was lower in comparison to the PG (135.35 ± 27.45) and LPG (140.84 ± 19.82), whose values were the highest among all six groups analyzed 8 weeks after nerve injury.

## 4. Discussion

The understanding of axonal regeneration has long been a subject of research in neuroscience and regenerative medicine [25]. The high incidence of these lesions and the lack of satisfactory results with surgical methods have increased the interest of researchers within interdisciplinary approaches [26]. Their studies have furthered the understanding of peripheral nerve recovery and similar problems in other regions of the nervous system [27].

The myelinated sciatic nerve had fibers of various diameters [28], and the exposure of the sciatic nerve (EG) did not lead to changes at both 4 and 8 weeks after the nerve injury. The treatments resulted in the improvement of the sciatic nerve, especially in the eighth week after injury. A decrease in the amount of myelin due to nerve crush [29] was observed in our study. Different crushing loads applied on the nerve (0.1 kg to 15 kg) resulted in Wallerian degeneration, and the severity of this degeneration is directly proportional to the applied load. Wallerian degeneration appears as large vacuoles, and during this process, endoneurial macrophages phagocytose and degenerate myelin [28]. An injury that leads to demyelination and failure to remyelinate consequently leads to axonal degeneration [29]. In the injured groups, Wallerian degeneration was also observed, with a loss of integrity of the internal constituents of nerves and a loss of myelin. The decrease in the densities of myelin and nerve fibers can be explained by the lack of remyelination, which generates axonal degeneration. The load of 15 kg was chosen due to its high capacity to damage of nerve fibers without disrupting the nerve layer envelopes, which causes an axonotmesis that rarely regenerates spontaneously up to 8 weeks after the nerve injury.

Axonal regeneration after nerve crush is slow; however, it is most successful when compared to axonal disconnection (neurotmesis) [30]. Even after 8 weeks, the spontaneous axonal recovery will not have the normal characteristics of fibers. This recovery occurs in approximately 24 weeks, and in many cases, it does not result in prelesion characteristics [31]. In this study, there was an improvement in the morphological and ultrastructural characteristics, even in animals that were not subjected to treatment (IG); however, this spontaneous recovery of the fibers until the 8th postoperative week did not restore preinjury features because the control group had a very distinct morphology from the IG.

The histological quantification assessed the total area of myelin in relation to the cross-sectional area of the sciatic nerve by quantifying pixels in digital images that were processed and transformed into a binary display (black and white). Density analysis using the quantification of myelin in pixels is unusual in studies of peripheral nerves; however, this type of analysis provided data about the recovery of this type of injury in a rapid, reproducible, and intelligible manner regarding the amount of myelin present in the nerve, which is dependent on the quality and speed of the pulse [29, 33, 34] and may become an interesting tool for this type of analysis. The density of blood capillaries is important because it relates to nutrition delivery and maintenance of tissue, particularly for repair after injury, and is crucial for the arrival of factors and nutrients that aid the recovery of this tissue [18].

Quantification revealed a histological improvement in sciatic nerve recovery after crush injury by employing treatments with LLLT and protein P1. After 4 weeks, the treated groups had improvement; however, it was not possible to choose the best treatment. After 8 weeks of injury, significant improvements were observed in the groups using the P1 protein (PG and LPG) and the results were close to the values of the control group. The morphometrics data also revealed that spontaneous regeneration was not achieved, as noted in the IG at 4 and 8 weeks after nerve damage, confirming the findings of Muratori et al. [32], who reported that after a period of 6 weeks, there was no spontaneous recovery and that the action of the treatments on the injured nerve was critical for obtaining the observed improvement.

The improvement in the morphological and morphometric characteristics following the use of LLLT after nerve crush was marked by a greater number of nerve fibers, myelinated fibers of larger diameter, and a higher amount of Schwann cells [35, 36]. In the present study, there was also an observed decrease in the amount of myelin after crushing (15 kgf), which is comparable with the density of nerve fibers diminished as reported by Oliveira et al. [28]. Based on these facts, we suggest the use of this therapy as a possible treatment for diseases and injuries that lead to a loss of myelin, for example, multiple sclerosis, which is prevalent in young adults and affects 2.5 million people [29, 37]. The number of capillaries present in injured nerves was not altered by irradiation with LLLT after 10 and 21 days [35]. Our results revealed no changes in the number of capillaries 4 weeks after the application of LLLT. The increased number of capillaries was observed only at 8 weeks after nerve injury. This result suggests that the change in the number of capillaries occurs after the first month of recovery from the application of LLLT.

The present study was the first to assess the ultrastructural and morphological changes that occur in a crushed nerve treated with the P1 protein. Thus, no data about the changes in injured tissue treated with this material were found in previously published studies. Our first observation reveals a very positive perspective on the use of this material for nerve crush injury because there were generally better recovery features obtained from using this protein.

The P1 protein showed great angiogenic potential in previous studies [17–19, 38]. In our study, 8 weeks after nerve injury, the treatments resulted in increased capillary density, with the highest values observed in the group that received the P1 protein (PG and PGL), suggesting that, in long term application, the P1 protein leads to increased vascularity of the region. 

## 5. Conclusion

In conclusion, treatment with LLLT and the P1 protein resulted in the improvement of peripheral nerve trauma recovery 4 and 8 weeks after injury. At 8 weeks after injury, the characteristics of the treated groups were closer resembled to those observed in the control group. In addition to higher capillary density, this suggests that there is a time-dependent characteristic of this type of recovery. In long-term recovery (8 weeks), the animals that received only the P1 protein generally had better recovery of crushed nerve, given the fact that the only application of LLLT with these proposed parameters not achieved the same morphological and morphometric results, and the association of the two treatments did not potentiate the recovery, suggesting that the improvement observed when the 2 treatments were applied mainly refers to protein P1. More studies are needed to improve the understanding of the recovery in other levels of analysis.

## Figures and Tables

**Table 1:** Low-level laser therapy parameters.

Power	30 mW
Intensity	0.75 W/cm^2^
Energy density	15 J/cm^2^
Wavelength	780 nm
Application time (per point)	20 s
Point of irradiation	3
Laser wave type	Continuous wave (CW)
Beam direction	Perpendicular to skin
Energy (per point)	0.6 J
Spot area	0.04 cm^2^
Sessions/frequency of irradiation	6/48 h

**Table 2:** Quantitative analyses 4 weeks after nerve injury.

Group	Nerve area (*µ*m^2^)	Myelin area (*µ*m^2^)	Ratio nerve/myelin area (%)	Capillaries/mm^2^
CG	506260.29	301583.14	60.28	86.03
EG	528856.68	298976.71	57.55	85.69
IG	547529.84	208071.30	32.37	74.45
LG	530146.08	215996.44	40.81	78.83
PG	622473.35	247001.80	43.72	77.80
LPG	649142.59	217904.45	36.64	98.16

**Table 3:** Quantitative analyses 8 weeks after nerve injury.

Group	Nerve area (*µ*m^2^)	Myelin area (*µ*m^2^)	Ratio nerve/myelin area (%)	Capillaries/mm^2^
CG	542780.48	258291.17	47.85	99.22
EG	601586.20	230101.07	40.60	87.60
IG	532432.67	136047.71	26.14	99.12
LG	474405.86	163314.33	35.89	116.59
PG	459164.22	186626.33	43.04	135.35
LPG	523248.26	225172.00	43.19	140.84
